# Quantifying and understanding reproductive allocation schedules in plants

**DOI:** 10.1002/ece3.1802

**Published:** 2015-11-07

**Authors:** Elizabeth Hedi Wenk, Daniel S. Falster

**Affiliations:** ^1^ Biological SciencesMacquarie UniversityNSW2109Australia

**Keywords:** Functional traits, growth strategy, iteroparous, life history, maximum height, reproductive allocation

## Abstract

A plant's reproductive allocation (RA) schedule describes the fraction of surplus energy allocated to reproduction as it increases in size. While theorists use RA schedules as the connection between life history and energy allocation, little is known about RA schedules in real vegetation. Here we review what is known about RA schedules for perennial plants using studies either directly quantifying RA or that collected data from which the shape of an RA schedule can be inferred. We also briefly review theoretical models describing factors by which variation in RA may arise. We identified 34 studies from which aspects of an RA schedule could be inferred. Within those, RA schedules varied considerably across species: some species abruptly shift all resources from growth to reproduction; most others gradually shift resources into reproduction, but under a variety of graded schedules. Available data indicate the maximum fraction of energy allocated to production ranges from 0.1 to 1 and that shorter lived species tend to have higher initial RA and increase their RA more quickly than do longer‐lived species. Overall, our findings indicate, little data exist about RA schedules in perennial plants. Available data suggest a wide range of schedules across species. Collection of more data on RA schedules would enable a tighter integration between observation and a variety of models predicting optimal energy allocation, plant growth rates, and biogeochemical cycles.

## Introduction

A primary goal of plant ecophysiological theory is to break down plant function into a common set of processes that identify strategic differences among individuals and species. By documenting links between individual tissues and allocation decisions on carbon uptake, growth, and mortality, plant ecology has moved decidedly toward a trait‐centric understanding of vegetation over the last 20 years (Reich et al. 1992; Westoby et al. 2002; Cornelissen et al. 2003; McGill et al. 2006; Chave et al. 2009; Wright et al. 2010). Given a common set of physiological rules describing plant construction and function, differences in growth strategy among species can increasingly be captured via a select number of functional traits (Falster et al. 2011). There is strong evidence for trade‐offs associated with leaf functioning, stem construction, plant hydraulics, and the division of reproductive effort into few large or many small seeds (Henery and Westoby 2001; Wright et al. 2004; Chave et al. 2009; Poorter et al. 2010). There also exists substantial and well‐documented variation among species in each of these traits (Westoby et al. 2002). However, we currently have a limited understanding of how species differ from one another in the amount of energy they allocate to reproduction, a key parameter in both optimal energy and plant growth models (Myers and Doyle 1983; Sibly et al. 1985; Miller et al. 2008; Fisher et al. 2010; Falster et al. 2011; Scheiter et al. 2013).

### Diversity of RA schedules

The partitioning of energy between reproduction and other activities throughout a plant's lifetime – such as growth, storage, and defense – is arguably the most fundamental component of its life history (Harper and Ogden 1970; Bazzaz et al. 2000). Here we refer to the fraction of surplus energy that is allocated to reproduction in a given period as reproductive allocation (RA), where surplus energy is that which remains after the costs of respiration and tissue turnover have been paid. As RA is expressed as a proportion of energy, it falls between 0 and 1. The change in *RA* with respect to size or age will be termed an *RA schedule*. We use surplus energy instead of net primary productivity as the energy pool to be subdivided, because for most perennial species, reproductive investment does not appear to come at the expense of existing tissues. This assumption is evident in the allometry of most trees, in which all size dimensions tend to increase over time. Use of “surplus energy” also aligns our study with many theoretical models, which invest in reproduction only after paying maintenance costs (e.g., early review by Kozlowski 1992) and plant growth models (e.g., papers by Thornley 1972; de Wit 1978; Mäkelä 1997). RA schedules then enact the outcome of a single fundamental trade‐off: the allocation of surplus energy between growth and reproduction. As such, they summarize essential elements of a plant's life history strategy: At what age do plants begin reproducing, what proportion of energy goes to reproduction, and how do plants moderate the proportion of energy they allocate to reproduction as they age? The follow‐on information is equally important, for energy not allocated to reproduction is used for growth, increasing the plants height and thereby its ability to outcompete neighbors for light (or other resources), hence increasing survival. From the perspective of other organisms, the RA schedule determines how gross primary productivity is allocated among fundamentally different tissue types, that is, leaves, woody tissues, flowers, fruits, and seeds, the eventual food stuffs at the base of terrestrial food webs.

**Figure 1:**
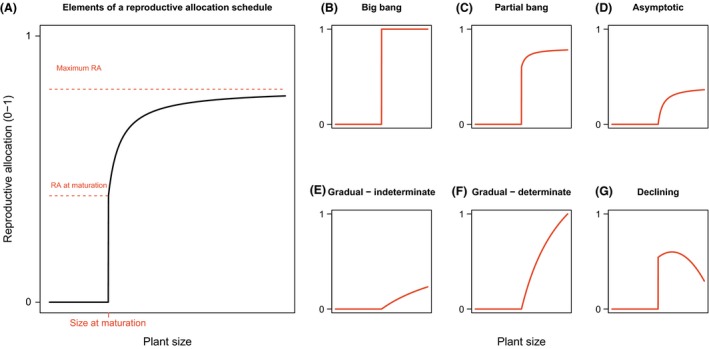
Classifying reproductive allocation schedules. Panel (A highlights elements of a schedule that can be quantified in their own right, while panels (B–G) illustrate alternative schedules.

**Figure 2:**
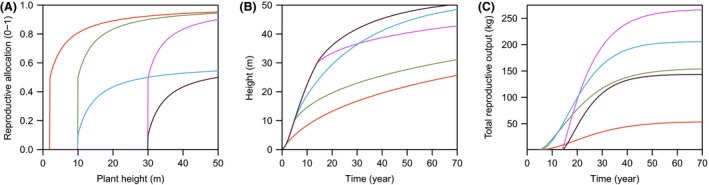
Reproductive allocation schedules influence growth rate, size, and seed output. Panel A. Using a generic model of plant growth (Falster et al. 2011), we simulated growth of five individual plants with different RA schedules. Panels (B–C) show how differences in height and lifetime reproductive output accumulate over time. Full details on model given in the supplied code (see end of methods).

The diversity of life history strategies observed across extant plant species suggests many different RA schedules might be expected (Fig. 1). The two most extreme RA schedules include a slow increase in RA across a plant's lifetime (a graded RA schedule) and an RA schedule where maximum RA is reached and vegetative growth ceases as soon as reproduction commences (a big bang schedule, indicating a switch from RA = 0 to RA≈1 across a single growing season) (Fig. 1). Big bang reproducers are also termed semelparous or monocarpic, a group that includes some annuals, several succulent shrubs, and at least a hundred trees (Young 2010; Thomas 2011) (Fig. 1, panel B). It is possible for a big bang species to cease growth and continue reproducing for several years, but most species die following a single large reproductive event (Young 2010). A graded RA schedule, also termed iteroparous or polycarpic, can be further divided into RA schedules we term partial bang, asymptotic, gradual, and declining, depending on how RA changes with size (Fig. 1C–G). Graded strategies are diverse, including RA schedules displaying early reproductive onset and high reproductive investment at the expense of growth and survival, as well as ones with a long period devoted entirely to growth followed by more modest reproductive output. Figure 2 highlights, using a simple plant growth model from Falster et al. 2011, how differences in RA schedule alone can drive differences in growth, seed production, and biomass allocation.

### Theoretical treatments of RA schedules

Theorists long ago adopted RA schedules as an elegant way to connect energy allocation with life history (e.g., Cole 1954; Myers and Doyle 1983; Kozłowski and Uchmanski 1987; Kozlowski 1992; Engen and Saether 1994; Miller et al. 2008). By incorporating the growth‐reproduction trade‐off, optimal energy allocation models identify the RA schedule that maximizes seed production across the plant's lifecycle under a given set of environmental conditions and for a given set of physiological traits (Kozlowski 1992). For instance, researchers have developed models that indicate how RA schedules vary with shifts in a variety of biotic and abiotic factors including tissue turnover (Pugliese and Kozlowski 1990), seed set (Miller et al. 2008), age‐specific mortality (Charnov and Schaffer 1973; Reznick and Endler 1982; Engen and Saether 1994), and environmental stochasticity (King and Roughgarden 1982; Gurney and Middleton 1996; Katsukawa et al. 2002).

### In a simple linear system, big bang is always optimal

The history of using optimal energy allocation to model RA schedules traces back to a seminal paper by Cole (1954). In his model, and subsequent similar ones, surplus energy can only go two places: to reproductive investment or vegetative production increasing the size of the plant. Moreover, there is a linear rate of energy conversion into these structures, so the trade‐offs between growth and reproduction are also linear. Optimal energy models that include only this direct linear trade‐off find that the complete cessation of growth with reproductive onset, a single reproductive episode, and subsequent death (i.e., the big bang strategy from Fig. 1, where RA switches from 0 to 1) is always optimal, because delayed reproduction when small and correspondingly greater growth leads to greater final reproductive output (Cole 1954; Kozlowski 1992; Perrin and Sibly 1993; Engen and Saether 1994). In these models, individuals with an iteroparous reproductive strategy (i.e., with an earlier start to reproduction, an RA <1, and multiple reproductive episodes) have a lower lifetime reproductive output than big bang reproducers. This is because with the iteroparous reproductive strategy, the onset of reproduction leads to decreased growth rates and a smaller adult size, resulting in lower lifetime surplus energy. The models predict that the size (or age) at reproduction of big bang reproducers shifts with factors such as growth rate, how increased size translates to increased reproductive output, and the probability of survival (Kozłowski and Wiegert 1987; Perrin and Sibly 1993); changing these parameters never causes the optimal RA schedule to shift away from big bang to a graded schedule. Yet the list of perennial semelparous plant species displaying a big bang strategy is relatively short, encompassing approximately 100 trees and some palms, yuccas, and giant rosette plants from alpine Africa (e.g., see Thomas 2011). This disconnect between theoretical prediction and observation has come to be known as Cole's Paradox (Charnov and Schaffer 1973) and has led researchers to search for mechanisms favoring a graded reproduction schedule.

### Nonlinear trade‐offs or environmental stochasticity promote graded allocation strategies

Cole's paradox has largely been resolved, as it is now known that a variety of other factors can shift the optimal energy allocation from “big bang” to a “graded” schedule. Specifically, models need to include either: (i) stochastic environmental conditions (King and Roughgarden 1982) or (ii) secondary functions influencing how efficiently energy allocated to different goals (growth, reproduction) is converted into different outcomes (increased vegetative size, seed production). It seems that if these conversion functions are nonlinear with respect to plant size, a graded allocation may be favored.

In one class of nonlinear trade‐offs, an auxiliary factor causes the cost of increased reproductive or vegetative investment to increase more (or less) steeply than is predicted from a linear relationship. As a first example, consider a function that describes how efficiently resources allocated to reproduction are converted into seeds. Studying cactus, Miller et al. (2008) showed that floral abortion rates due to insect attack increased linearly with RA. In other words, as RA increases, the cost of creating a seed increases, such that the cacti are selected to have lower RA and earlier reproduction than would be expected from direct costs of reproduction alone. A second example, Iwasa and Cohen's model (1989) showed that declining photosynthetic rates with size, a trend detected in several empirical studies (Niinemets 2002; Thomas 2010), led to a graded RA schedule. Third, many models, often backed up with data from fish or marine invertebrates, have shown that if mortality decreases with age or size, it benefits an individual to grow for longer and then begin reproducing at a low level – a graded RA schedule (Murphy 1968; Charnov and Schaffer 1973; Reznick and Endler 1982; Kozłowski and Uchmanski 1987; Engen and Saether 1994). Overall, optimal energy models show that a great diversity of graded RA schedules is possible, and that as suggested, both fundamental life history traits (mortality, fecundity) and functional trait values (photosynthetic rate, leaf life span, growth rates) could affect the shape of the RA schedule.

### Need for empirical data

While the outcomes of the many optimal energy models show that RA schedules shift depending on a plant's collection of life history and physiological traits, there is little empirical data to test the outcomes of these models. Widespread collection of empirical data has been limited due to the effort required to accurately determine the many sinks for surplus energy, including growth, storage, defense, and reproduction. In particular, very few data on lifetime reproductive allocation exist for long‐lived species, due to the impracticalities of assessing reproductive output across an individual tree's lifetime.

In this study, our first aim is to review the available empirical RA schedules in nonclonal, woody plants with bisexual flowers. We present a summary of empirical data for the handful of studies quantifying complete RA schedules, as well as some data sets that include only particular features of an RA schedule, such as the shape of the curve. Despite several reviews about elements of plant reproduction (Bazzaz et al. 2000; Obeso 2002; Moles et al. 2004; Weiner et al. 2009; Thomas 2011), none have explicitly focused on RA schedules or the integration between empirical data and the outcome of theoretical models. This review focuses on perennial species, for recent work has established a framework for investigating reproductive output (RO) in annuals (Weiner et al. 2009). Studying reproductive investment in perennial species is more challenging, but very relevant, as these species are the dominant contributors to woody plant biomass worldwide. We predict that species will display a diversity of RA schedules and that shorter lived species will have relatively high RA and reach their maximum RA more quickly than do longer‐lived species. Second, we summarize studies that compared RA or RA schedules across individuals, populations, or species growing under different disturbance regimes or with different resource availabilities, and hence give insight on what environmental, life history, or functional traits might alter either RA at a given age or size or the entire RA schedule. We expect 1) that individuals in poor resource environments will postpone reproduction and have lower annual RA and 2) that individuals in disturbance‐prone environments will begin reproducing at younger ages and have higher annual RA. In the discussion, we compare the information gleaned from our compilation of RA schedules with that provided by measures of RO and the research questions each method best address.

## Methods

### Defining and quantifying reproductive allocation schedules

**Figure 3:**
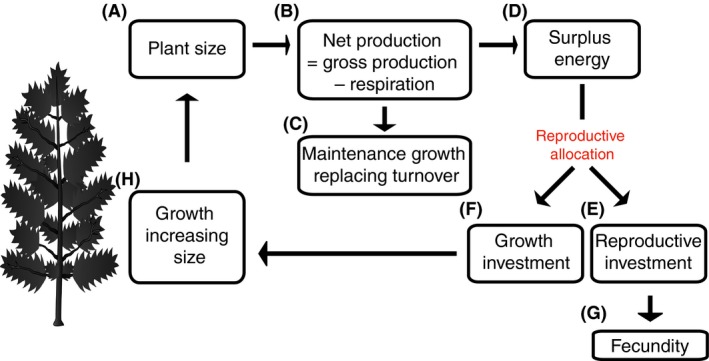
Energy flow within a plant, showing how a given quantity of surplus energy is divided between reproductive investment and growth. Note that total vegetative growth includes maintenance growth, replacing parts lost via tissue turnover, and new growth leading to a net increase in size, termed “growth beyond replacement” in the text.

A conceptual outline of the energy budget for a plant illustrates how RA is calculated (Fig. 3). To calculate the amount of energy allocated to growth, it is necessary to distinguish between growth that replaces lost tissues and growth that increases the size of the plant. Beginning at Figure 3A, consider that a plant of a given size and with a given collection of functional traits has a given gross primary production (GPP) and respiration costs. Subtracting respiration from GPP yields net primary production (NPP). Some of this NPP will be used to replace lost or shed tissue (Fig. 3C), with the remainder designated as “surplus energy” (Fig. 3D). (Energy can also be allocated to storage or defense, but for simplicity these are not included. If surplus energy is allocated to storage – and hence unmeasured – surplus energy will be underestimated and RA will be an overestimate.) Note that total growth on the plant in a given year is not one of the boxes, because it represents a combination of energy used to replace lost tissues, that is, the portion of NPP a plant used to maintain current size, and the portion of surplus energy allocated to growing to a bigger size during the survey period.

To properly quantify an RA schedule, one must measure all the energy allocated to growth and reproduction over time. In principle, an RA schedule concerns the instantaneous fraction of surplus energy allocated among growth and reproduction. In reality, RA should be measured over longer time periods, because growth and reproduction often occur at different points during the growing season. The energy budget is therefore typically tabulated on a per year basis. Some species have inconsistent year‐to‐year reproductive output, termed masting. For these species, the energy budget must be tabulated across a mast year and the number of nonmast years that follow. The weight of dry biomass is the most commonly used proxy for “energy,” but the kilojoules energy contained in the biomass or the mass of a specific limiting element are valid alternatives. It is important that the same energy units be used for both reproductive and vegetative material.

Reproductive investment should be measured over an entire reproductive cycle and include energy invested both in seed and accessory tissues, the latter termed accessory costs. Accessory costs include the construction of prepollination (flower, nectar, and pollen) and postpollination (packaging, protective and dispersal tissues; aborted ovules) floral parts. Total accessory costs are highly variable and can be as much as 99% or as little as 15% of reproductive energy investment (Table 1).

**Table 1:** Compilation of data from studies measuring reproductive accessory costs. Values give the range of each accessory cost as a percentage, with the mean shown in brackets. Prepollination costs are both those required to construct the inflorescence, as well as nectar production to entice pollinators, and pollen production. Inflorescence costs include support structures (receptacle, peduncle) and floral parts (sepals, petals, stamens, stigma, ovary, ovules). The postpollination cost of aborted ovules includes aborted immature seeds at all stages. Packaging, protective, and dispersal costs include abiotic dispersal structures, tissue that attracts animal dispersers, and enlarged receptacles. Finally, seed cost is the actual cost of the seed, independent of the rest of the fruiting structure

Authors	Species or life‐form	Number of species	Prepollination costs			Postpollination costs			Seed costs (%)
			Inflorescence (%)	Nectar production (%)	Pollen production (%)	Aborted ovules (%)	Packaging, protective and dispersal costs (%)	Total accessory costs (%)	
Lord and Westoby (2006)	Many species and life‐forms	14	0.5–63 (15.7)	Not measured	Not measured	0.6–72 (12.9)	0.7–94 (43.2)	33.4–96.1 (71.8)	4–67 (28.2)
Henery and Westoby (2001)	Serotineous Proteaceae	10	Not measured	–	–	Included in next category	90–99 (97.7)	90–99 (97.7)	5–55 (2.3)
Henery and Westoby (2001)	Woodland and heathland perennials	37	Not measured	–	–	Included in next category	15–95 (70)	15–95 (70)	5–85 (30)
Greene and Johnson (1994)	Trees	17	Not measured	–	–	Data not provided	Data not provided	23–97 (69)	3–77 (31)
Chen et al. (2010)	Subtropical woody dicots	62	Not measured	–	–	Included in next category	15–98 (47)	15–98 (47)	2–85 (53)
Ashman (1994)	*Sidalcea oregana*, hermaphrodites	1	60	N/A	4	Not measured	<1, so ignored	64	36

To calculate the investment in growth, one must determine how much bigger the plant is, relative to a year earlier. Unless you are able to follow a single plant through its life, you must find individuals of different sizes, preferably of known age, on which to measure RA. These individuals should be growing under similar environmental conditions and in a similar community of species. One approach to estimating a complete RA schedule for long‐lived species is to pick a known chronosequence, as is available with plantation trees and in locations with a known disturbance (and germination) history (Zammit and Zedler 1993; Cleary et al. 2008; Genet et al. 2010). Combining RA measurements from plants across a range of sizes yields an RA schedule; a curve showing how an individual's relative investment in reproduction shifts with plant size or age (Fig. 1). We have focused on size‐related patterns, as size has been shown to have a greater influence on RA than age (Herrera 1991; Pino et al. 2002). In particular, size is the primary factor determining the onset of reproduction in competitive environments (Pino et al. 2002).

### Literature

Here we review what can be learned about RA data from existing studies on 34 populations, representing 32 species. These are the only studies we found in the literature that include data either on how RA changes with size (or age) or that compare RA across populations or closely related species. We searched widely in the literature using both Web of Science and Google Scholar for studies that had measured reproductive investment at multiple ages, across different resource environments or under different disturbance regimes. Some studies used a known chronosequence, some followed the same individuals (or population) across multiple years, and yet others used co‐occurring individuals of different sizes to construct a RA schedule. Additional studies report measures of RO, proxies for RA, such as flowering intensity (e.g., Herrera and Jovani 2010) or number of reproductive modules (e.g., Miller et al. 2008), but not actual biomass or energy allocation to reproduction. Ideally, RA values were available for individuals at multiple sizes (or ages), such that a RA schedule could be plotted. Knowing RA at reproductive onset and 2–3 later time points is sufficient to predict the shape of the RA schedule, but of course more data points increased the precision with which the RA schedule could be drawn. We included studies from which the shape of the RA schedule can be estimated, even if absolute RA values cannot be calculated. The categorization of RA schedule types (Fig. 1) is based on a visual assessment, as data are insufficient for a statistical classification. Studies solely reporting plots of reproductive biomass against plant size have not been included as they have been thoroughly reviewed recently (Weiner et al. 2009; Thomas 2011) and do not provide any means of determining whether a plant with a large reproductive capacity has a high rate of mass production or large allocation to reproduction. Most of the studies included have not themselves explicitly plotted RA schedules, but instead provide data that can be used to quantify RA schedules (see [Link] for details). The studies comparing RA in populations or species subjected to different resource conditions or disturbance regimes do not have data on different sized individuals; instead, these data indicate how these variables might shift certain parts of an RA schedule.

Based on published information, RA was calculated as the proportion of total surplus energy, on a per time basis, allocated to reproduction. One year (or one growing season) is the commonly used time interval. Energy units used are per gram dry mass or kilojoules (determined by burning the samples). Total surplus energy is calculated as the sum of RO, “growth beyond replacement,” as defined in Figure 3, energy stored underground, and energy allocated to defense. RO is the sum total of all types of reproductive investment: flowers, nectar, aborted fruit, mature fruit, and vegetative structures associated only with flowering. It is noted in Table 1 when studies report total new growth, not growth beyond replacement; using total new growth instead of “growth beyond replacement” overestimates surplus energy and underestimates RA. Very few studies consider energy stored underground and energy allocated to defense. When available, these are summed with growth, otherwise this pool is ignored (set to zero). If growth beyond replacement is not directly reported, it is estimated from data on increase in stem diameter and increase in leaf area. RA is then calculated and plotted against plant size (or age) to determine the shape of the RA schedule. Unfortunately, most studies report data for only some reproductive components, usually ignoring shed accessory tissues. The missing reproductive costs are thus not included in our analysis, which will cause RA to be underestimated.

Individual components of an RA schedule are presented in Table 2 and discussed below. They include the shape of the RA schedule, RA at maturation, maximum RA, and size at maturation. For the following studies, the numbers presented in Table 2 were taken directly from the published articles: Pitelka 1977; Pritts and Hancock 1983; Oyama 1990; Alvarez‐Buylla and Martinez‐Ramos 1992; Comps et al. 1994; Ehlers and Olesen 2004; Poorter et al. 2005; Read et al. 2006, 2008; Miller et al. 2008. For the remaining studies, we calculated RA schedules using published data (see [Link] for details).

**Table 2:** A compilation of available data on reproductive allocation schedules. The shape of the curve is given for all studies, while more precise numbers including RA at the onset of reproduction (threshold RA) and maximum RA are given for the subset of species with available data. The method for determining the plant growth used to calculate RA is given as “allometric equation” indicating an equation was derived to correlate a diameter with a specific plant mass or “harvest” indicating the plants were collected and weighed at the end of the study

Growth from	Species name	Habitat	Size measure	Growth method	Shape of curve	Threshold RA	RA currency	Maximum RA	RA bias	Size at maturation	Reference
Cactus	*Opuntia inbricata*	Desert	–	–	Asymptotic	–	–	–		–	Miller et al. (2008)
Herb	*Corydalis*	Temperate, understorey	Tuber volume (cm^3^)	Allometric equation	Partial bang	–	–	–		–	Ehlers and Olesen (2004)
Herb	*Lupinus variicolor*	Stressful	Height (m)	Harvest	Partial bang	0.18	Joules	0.22	None	–	Pitelka (1977)
Herb	*Solidago pauciflosculosa*	Temperate	Dry weight (g)	Harvest	Asymptotic	0.16	Dry weight	Lifetime RA = 0.3	Under	6	Pritts and Hancock (1983)
Palm	*Astrocaryum mexicanum*	Tropical, understorey	Dry weight (kg)	Allometric equation	Asymptotic	0.05	Dry weight	0.70	None	2	Piñero et al. (1982)
Palm	*Chamaedorea tepejilote*	Tropical, understorey	Height (m)	–	Asymptotic	–	–	–		0.5	Oyama (1990)
Palm	*Rhopalostylis sapida* (Nikau palm)	Temperate	Height (m)	Frond counts and allometric equation	Asymptotic	0.08	Joules	0.56	Under	4	Enright (1985)
Shrub	*Lupinus arboreus*	Early successional	Height (m)	Harvest	Partial bang	0.21	Joules	0.26	None	–	Pitelka (1977)
Shrub	*Vaccinium corymbosum*	Temperate, understorey	Dry weight (g)	Harvest	Declining	0.25	Dry weight	0.53	Under	–	Pritts and Hancock (1985)
Tree	*Abies mariesii*	Temperate, high altitude	Height (m)	Allometric equation	Declining	–	Dry weight	–		2.1	Sakai et al. (2003)
Tree	*Abies mariesii*	Temperate, low altitude	Height (m)	Allometric equation	Asymptotic	–	Dry weight	–		4.6	Sakai et al. (2003)
Tree	*Abies mariesii*	Temperate, mid altitude	Height (m)	Allometric equation	Gradual ‐ indeterminate	–	Dry weight	–		3.2	Sakai et al. (2003)
Tree	*Abies veitchii*	Temperate	Height (m)	Allometric equation	Declining	0.04	Dry weight	0.06^a^	Possible	4	Kohyama (1982)
Tree	*Cerberiopsis candelabra*	Temperate	–	–	Big bang	1	–	1		–	Read et al. (2006, 2008)
Tree	*Cercropia obtusifolia*	Tropical, pioneer	Basal diameter (cm)	–	Asymptotic	–	–	–		10	Alvarez‐Buylla and Martinez‐Ramos (1992)
Tree	*Fagus sylvatica*	Temperate	Height (m)	Allometric equation	Asymptotic	0.09	Dry weight	0.43	Under, over	15	Genet et al. (2010)
Tree	*Fagus sylvatica*	Temperate	–	Harvest of shoots	Gradual ‐ indeterminate	–	–	–		–	Comps et al. (1994)
Tree	*Lindera erythrocarpa*	Temperate	Height (m)	Allometric equation	Gradual ‐ indeterminate	0.009 (0.004^a^)	Dry weight	0.17 (0.07^a^)	None	10	Hirayama et al. (2004)
Tree	*Quercus acuta*	Temperate	Height (m)	Allometric equation	Gradual ‐ indeterminate	0.06	Dry weight	0.22	None	14	Hirayama et al. (2008)
Tree	*Quercus salicina*	Temperate	Height (m)	Allometric equation	Unknown: flat across range		Dry weight	0.42	None		Hirayama et al. (2008)
Tree	*Quercus sessilifolia*	Temperate	Height (m)	Allometric equation	Gradual ‐ indeterminate	0.03	Dry weight	0.63	None	15	Hirayama et al. (2008)
Tree	*Tachigali vasquezii*	Temperate	–	–	Big bang	1	–	1		–	Poorter et al. (2005)

a Total yearly growth, not just growth beyond replacement.

### Reproducibility

All analyses were conducted with R software (R Core Team 2014). The code and data for producing all figures in this study is available at https://github.com/dfalster/Wenk_RA_review.

## Review of Empirical Data

### Lifetime reproductive allocation schedule

The species sampled exhibit an enormous variety of reproductive strategies, from truly big bang species (Fig. 1B, Table 2) to a great diversity of graded reproduction schedules (Fig. 1C–G, Table 2). We included only two species with big bang RA schedules; all others exhibit one of the graded RA schedules. Three species, including most perennial herbaceous species studied, ramp up to their maximum RA within a few years of reproductive onset (Pitelka 1977; Ehlers and Olesen 2004) and are classified as “partial bang” (Fig. 1B). Eight species show a more gradual increase in RA, but still reach a definite plateau, the “asymptotic” type in Fig. 1D (Piñero et al. 1982; Oyama 1990; Alvarez‐Buylla and Martinez‐Ramos 1992; Genet et al. 2010). Five of the longest lived species, including both evergreen and deciduous temperate trees, continue to increase RA throughout their lives, never reaching an obvious asymptote (Comps et al. 1994; Hirayama et al. 2004, 2008), and are therefore labeled “gradual‐indeterminate” (Fig. 1E). No species had an RA schedule we visually categorized as “gradual‐determinate” (Fig. 1F). This collection of RA schedules matched our expectations that some species displayed few years of relatively high RA and others many years of mostly lower RA. Faster growth allowed a monocarpic species *Tachigali vasquezii* to reach a large size and reproductive maturity more quickly than co‐occurring iteroparous species; that is, faster growth allowed the onset of reproduction to be advanced (Poorter et al. 2005).

In most of the studies considered, the maximum RA achieved is maintained until the end of life, in agreement with evolutionary theory predicting increasing or stable RA until death (Roff 2002; Thomas 2011). However, there are three species, *Vaccinium corymbosum* (Pritts and Hancock 1985), *Abies veitchii* (Kohyama 1982), and high elevation populations of *Abies mariesii* (Sakai et al. 2003), where RA decreases late in life and thus exhibit a “declining” RA schedule (Fig. 1G, Table 2).

### Reproductive allocation at maturation

Threshold reproductive allocation was reported for 15 species and populations. Long‐lived iteroparous species usually initially have very low RA values, such as 0.05 for *Rhopalostylis sapida* (Nikau Palm) (Enright 1985) and 0.08 for beech (Genet et al. 2010) (Table 2). By contrast, shorter lived species can have quite high RA values the year they commence reproduction, such as 0.25 for *Vaccinium corymbosum* (Pritts and Hancock 1985) and 0.18 for *Lupinus variicolor* (Pitelka 1977) (Table 2). Two semelparous perennial species, ones with a big bang schedule where they instantaneously reach RA = 1, are included in Table 2. Several hundred additional species are known to have this life history (Young 1984, 2010; Klinkhamer et al. 1997; Thomas 2011).

### Maximum reproductive allocation

Thirteen of the studies reported maximum RA. For semelparous species, such as *Tachigali vasquezii* and *Cerberiopsis candelabra,* it is always close to 1 (Poorter et al. 2005; Read et al. 2006). Iteroparous species usually have a maximum RA between 0.4 and 0.7 (Table 2), although values as low as 0.1 have been recorded in an alpine community (Hemborg and Karlsson 1998). Long‐lived iteroparous species are expected to have lower maximum RA than shorter lived species, as they are diverting more resources to survival, both in the form of more decay and herbivore resistant leaves and stems and other defense measures. These species compensate for a lower RA by having more seasons of reproductive output. However, no clear trend in longevity versus maximum RA is noted among the studies in Table 2, with the highest RA, 0.70, recorded in a temperate palm that lives for more than 250 years.

### Shifts in reproductive allocation with disturbance frequency or resource availability

Comparisons across species or populations that are subject to different environmental conditions have identified certain RA schedule components that recurrently co‐vary, suggesting convergent adaptation. In each case, the two populations (or species) grow either in locations that differ in resource availability or in disturbance frequency (effecting mortality), with resultant shifts in RA schedule components. Species or populations with smaller threshold size or earlier maturation, generally have higher RA, supporting traditional life history theory that weedy species have higher fecundity (Stearns 1992; Table 3). Higher mortality is also correlated with this fast‐growth strategy, suggesting that the aforementioned traits compensate for having fewer years to reproduce. Lower resource availability is recurrently correlated with lower RA and delayed maturation. Of these studies, only Sakai et al. (2003) have sufficient data to plot complete RA schedules (see Table 3), with the other studies only providing data on portions of the RA schedules such as size at reproductive onset, initial RA, or maximum RA.

**Table 3:** (a) Studies showing a correlation across populations or closely related species between RA or threshold size (or age) and a demographic parameter or plant dimensions. The ecological explanation given by the authors is included. (b) Summary of number of studies showing increase and decrease in RA or timing of reproduction with changes in mortality or resource availability

(a)				
Study unit	Species	Observed correlation	Ecological explanation	Reference
Populations	*Attalea speciosa*	Shadier environment → Larger threshold size	Individuals in lower resource environments must be bigger before they can afford to allocate energy to reproduction.	Barot et al. (2005)
Populations	*Drosera intermedia*	Higher adult mortality → Higher RA, in some environments	Individuals with fewer years to reproduce must allocate more energy to reproduction.	de Ridder and Dhondt (1992a,b)
Species	*4 alpine and subalpine species*	Higher elevation (lower resource environment) → Lower RA	Species in lower resource environments can afford to invest less energy in reproduction.	Hemborg and Karlsson (1998)
Species	*3 Pinguicula* species	Higher adult mortality → Higher RA	Individuals with fewer years to reproduce must allocate more energy to reproduction.	Karlsson et al. 1990; Svensson et al. (1993)
Populations	*Verbascum thapsus*	Higher mortality → Smaller threshold size	Individuals in environments that become inhospitable more quickly have fewer years to reproduce and must begin reproducing at smaller sizes.	Reinartz (1984)
Populations	*Abies mariesii*	Higher mortality → Earlier maturation, higher RA	Individuals in environments with greater mortality must begin reproducing earlier and must allocate more energy to reproduction.	Sakai et al. (2003)
Populations	*Pinus pinaster*	Less favorable environment (PCA of multiple climatic features) → Higher RA, smaller threshold size (with respect to female function)	Individuals in overall unfavorable environments must begin reproducing earlier and must allocate more energy to reproduction.	Santos‐del‐Blanco et al. (2010, 2012)
Populations	*Cynoglossum officinale*	Lower growth rates, higher mortality → Smaller threshold size	Individuals in overall unfavorable environments must begin reproducing at smaller sizes.	Wesselingh et al. (1997)
Species	Grasses	Poor resource environments –> Lower RA, delayed maturation	Species in lower resource environments must be bigger before they can afford to allocate energy to reproduction and even then allocate less energy to reproduction.	Wilson and Thompson (1989)

(b)			
		Higher mortality	Fewer resources
RA	Higher	4	0
	Lower	0	2
Timing of reproduction	Earlier/smaller size	4	1
	Delayed/larger size	0	2

## Discussion

Using RA schedules to compare reproductive strategies across species (or populations) distinguishes between energy allocated to fundamentally different tissue types and thus links to a key physiological trade‐off in an organism's functioning and life history. Plants that allocate more of their surplus energy to reproduction release more seed in a given year, but grow less. This potentially exposes them to increased competition, as others that defer reproductive investment progressively overtop the plant. Yet, despite the long‐recognized importance of RA schedules as a key life history trait (Harper and Ogden 1970) and the many optimal energy models that have investigated what causes RA schedules to shift, remarkably few RA schedules have been quantified. The limited data available do however suggest that plants display an enormous diversity of RA strategies, ranging from the “big bang” strategy displayed by semelparous species to a variety of graded reproduction strategies, with maximum RA in iteroparous species ranging from 0.2 to 0.7 (Table 2). Studies that compared RA (at a single age or size) across populations (or species) with different resource availability or disturbance frequency (Table 3) suggest populations (or species) that are short lived have earlier maturation and rapidly increase RA after maturation. In contrast, lower mortality and later maturation would be associated with a very gradual increase in RA and a slow approach to maximum height (i.e., gradual‐indeterminate or asymptotic strategy).

These data support analyses of life table data: higher resource or high disturbance environments tend to be home to individuals (and populations and species) with low survival, high fecundity, high growth rates, early reproductive maturity, and short life span, versus individuals with the opposite collection of trait values (Bender et al. 2000; Forbis and Doak 2004; Franco and Silvertown 2004; Garcia et al. 2008; Burns et al. 2010). Optimal energy models likewise show increased environmental stochasticity leads to earlier reproduction (King and Roughgarden 1982; Gurney and Middleton 1996; Katsukawa et al. 2002). Different functional trait values, including growth rates and energy investment into specific tissues, should also influence RA schedules, but more data are required to make trait‐based groupings. In addition, statistical comparisons of RA schedules across species can be made if researchers converge on more similar methods, as many methods were used to determine the RA schedules summarized here.

### Alternative measures of reproductive function

Much research has focused on components of reproductive function, including measures of reproductive output (RO; Henery and Westoby 2001; Niklas and Enquist 2003; Weiner et al. 2009), relationships between reproductive output versus vegetative mass (RV curves; Weiner et al. 2009), a species' maximum height (Wright et al. 2010; Cornwell et al. 2014), and relative size at onset of maturity (RSOM; Wright et al. 2005; Falster and Westoby 2005; Thomas 2011). We now consider the value of these metrics, versus RA, in quantifying reproductive patterns and their relative benefits for addressing different research questions.

**Figure 4:**
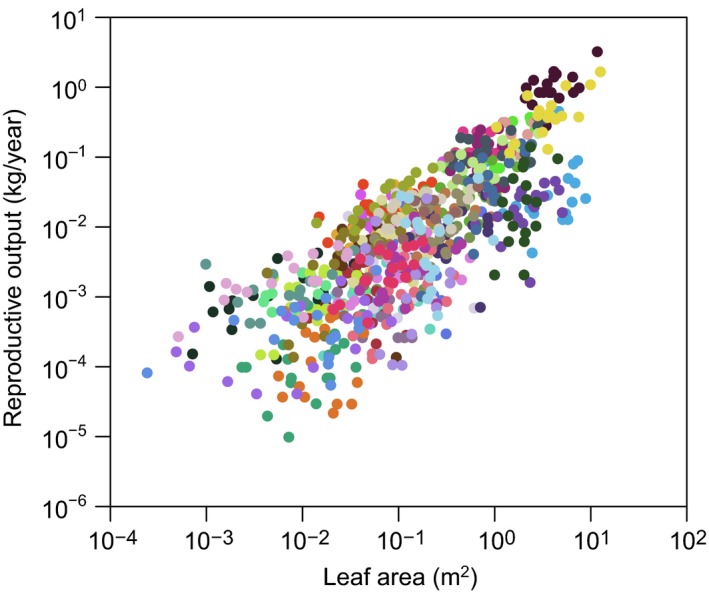
Variation in reproductive output with size within populations for 47 co‐occurring species. Data are from Henery and Westoby (2001). Fruiting and seed production data were collected for 47 woody perennial species over a period of 1 year in Ku‐ring‐gai Chase National Park, Australia. In each species, annual fruit production data for six randomly selected reproductively mature individuals per species at each site were collected over a period of 12 months as the fruit matured. Each dot represents an individual; species are distinguished by colors.

Reproductive output is the measure of seed production per unit time (either in numbers or units mass). To first order, plants increase reproductive output by growing larger as the productive capacity of a plant increases along with its total leaf area (Müller et al. 2000; Niklas and Enquist 2003; Weiner et al. 2009; Fig. 4). The relationship between plant size and RO can be examined by constructing a log–log regression of cumulative lifetime RO against vegetative size – an “RV curve” (Samson and Werk 1986; Klinkhamer et al. 1992; Bonser and Aarssen 2009; Weiner et al. 2009). An RV curve allows one to estimate the lifetime RO of an individual of a given size, an important metric for a diversity of plant population biology, agricultural, and conservation biology research questions. In contrast, an RA schedule only informs us of the amount of energy invested in reproduction, and thus, how many offspring are produced, if growth rates are also known, leading to criticism that using allocation ratios to measure changes in reproductive output across a plant's lifetime is limiting (Jasienski and Bazzaz 1999; Müller et al. 2000; Weiner 2004). If the RV curve is known for a species, the size of all individuals in a population can rapidly be estimated and the total RO calculated. A RV curve is equally applicable for high and low resource environments and different population densities, because differences in plant size lead to corresponding shifts in RO.

For other research questions however, RA schedules add information: they frame reproductive investment as a trade‐off to growth and separate the effects of large plant size and large reproductive investment on RO. RA schedules embody how increased allocation to reproduction impacts growth in a given year (or growing season) and therefore affects both the competitive interactions between species in a community and individual survival. One species could grow fast and have early RO, while another could have slower growth and delayed RO; both could have similar RV curves, but very different life spans, for the species diverting resources to reproduction at a smaller size is likely to be outcompeted for light (or water or nutrients) by co‐occurring species and be shorter lived.

RA schedules are also important for dissecting the contribution of yearly growth versus preexisting size to RO; RV curves and plots of the ratio of RO to plant biomass versus plant size provide no data on how much a plant grows in a given year, just how large it is. Consider Figure 4 that presents data on annual RO in relation to size for 47 coexisting plant species. It shows that for most species, RO increases with size, but that species differ by at least two orders of magnitude in the amount of production at any given size. Do such differences reflect different levels of photosynthetic productivity? Or do they indicate different levels of allocation to seed production? If one knew both the plant's RA schedule and its growth rates, one could separate the effects of RA and productive capacity on RO. Two plants of a given size could have identical RO, but one would have higher productive capacity and a lower RA and a second plant could have the reverse. As plants age their pool of surplus energy may begin to plateau or even decrease, both through declining photosynthetic capacity (Niinemets 2002; Thomas 2010) and increasing tissue replacement costs. Plots of RO against plant size indicate RE approaches an asymptote. Yet from the limited empirical data (Table 2) and optimal energy theory we know that RA may not be constant as a plant increases in size. Indeed, unlike RE, RA often continues to increase across an individual's life and the rate of increase in RA with size varies with life history.

Maximum height and RSOM, the ratio of threshold size (size at reproductive onset) to maximum size, are two other metrics used to assess the trade‐off between growth and reproduction. Like RA, they are based on the assertion that allocation to reproduction impacts growth (Thomas 1996; Davies and Ashton 1999). RSOM is used to summarize the trade‐off between continued faster growth rates and greater maximum height versus earlier reproduction, curtailed growth, and lower maximum height (Thomas 2011). The premise for using maximum height is that a species with a greater maximum height has delayed diverting energy to reproduction for longer and hence maintained a greater growth rate for longer during development (Turner 2001; Westoby et al. 2002). The tallest species in a community are predicted to be the long‐lived, later reproducing species that allocate less of their yearly energy to reproduction. Greater maximum height was correlated with higher potential growth rate in adults in tropical forests (Wright et al. 2010), but this study does not include any data on reproductive output. The advantage of using maximum height as a proxy for reproductive allocation is that it is easy to measure: Data now exist for over 20,000 species (Cornwell et al. 2014). The main problem with maximum height is that it quantifies the outcome of both demographic luck and a whole host of individual trade‐offs, not just the RA trade‐off. Moreover, the nature of all these trade‐offs may shift with age and/or across its geographic range. As is shown in Figure 2, different RA schedules can yield the same final maximum height, but with different growth rates along the way, leading to different competitive interactions. Thus, both RSOM and maximum height might be more usefully seen as outcomes of an RA schedule rather than predictors of it.

While the above‐mentioned measures of reproductive function may be easier to quantify across large numbers of species, they cannot substitute for a complete RA schedule. In particular, none of those measures directly captures the seasonal or yearly decision faced by the plant of where to allocate surplus energy, making them difficult to incorporate into process‐based models of vegetation dynamics (e.g., Fisher et al. 2010; Falster et al. 2011; Scheiter et al. 2013). Neither RV curves nor current season RO can be incorporated into such models, because both only capture the output of energy allocation, rather than the process itself. In contrast, an RA schedule has a direct process‐based definition: it specifies the proportion of energy allocated to reproduction as a fraction of the total energy available, at each size or age.

### Considerations when measuring reproductive allocation schedules

Overall, we advocate for greater measurement of RA schedules. Given RA schedules have been called the measure of greatest interest for life history comparisons (Harper and Ogden 1970; Bazzaz et al. 2000), we are surprised by just how little data exist. As described above, we are aware of the variety of challenges that exist to accurately collect this data, including accounting for shed tissue, all reproductive costs, and the yearly increase in size across multiple sizes and/or ages. In addition to these methodological difficulties, we will briefly introduce some other intricacies.

There has been debate as to the appropriate currency for measuring energy allocation. Almost all studies use dry weight or calorie content (joules) as their currency. Ashman (1994), whose study had one of the most complete point measures of RA, showed that carbon content is an inferior predictor of underlying trade‐offs compared to nitrogen and phosphorus content, although the general patterns of allocation did not shift with currency. Other studies have found all currencies equally good (Reekie and Bazzaz 1987; Hemborg and Karlsson 1998), supporting the theory that a plant is simultaneously limited by many resources (Chapin et al. 1987).

A complicating factor in determining RA schedules (or any plot showing yearly reproductive investment), is that many species do not have consistent year‐to‐year reproductive output (Kelly and Sork 2002; Smith and Samach 2013). Indeed, many species, including ones represented in 3 of the studies included in Table 2, mast, indicating they have years with far‐above average reproductive investment, following by one or more years with near‐zero reproduction. For these species, reproductive investment must be the average of a mast year and the relative number of nonmast years observed in that species.

A topic we have not seen discussed in the RA allocation literature is how to account for the transition of sapwood to heartwood. If functionally dead heartwood were considered part of the shed tissue pool, far more of a plant's annual energy production would be spent replacing this lost tissue, decreasing surplus energy and greatly increasing estimates of apparent RA for all plants, especially as they approach the end of life. It may even result in more iteroparous species actually approaching RA = 1 in old age, as is predicted in many models.

A recent model, however, suggests that reproductive restraint can be beneficial late in life, if it allows an individual to survive for an additional season and have even a few additional offspring (McNamara et al. 2009). An alternative hypothesis put forward is that species that can be long‐lived may none‐the‐less benefit from high RA early in life, because the patch environment will be most favorable to the species' recruitment closer to the time the individual itself germinated (Kohyama 1982; Nakashizuka et al. 1997; Ehlers and Olesen 2004). Under this scenario, the species may quickly reach a high RA and later as the patch environment degrades display reproductive restraint if there is a small probability individuals can survive until the patch environment is again ideal for recruitment. This argument most obviously applies to understory species increasingly shaded by a canopy (Pritts and Hancock 1985; Ehlers and Olesen 2004), but was also proposed by Kohyama (1982) to explain decreasing RA with stand age in a canopy tree. Alternatively, these patterns may result from incomplete measurements, such as underestimating tissue turnover rates (Fig. 3). At this point, there is just too little data to draw many general conclusions, or assess whether methods of data collection are influencing our results.

### Utility of reproductive allocation schedules and future directions

Over 40 years ago, Harper and Ogden (1970) recognized the intrinsic value for RA in understanding plant function, stating that “Ideally a measure of reproductive effort would involve the determination of starting capital, gross production, and that fraction which is output in the form of propagules.” Energy invested in reproduction reduces the pool of energy available for plant growth – either growth in height, maintaining access to light or growth in leaf area, and hence photosynthetic gain. As such, we and others have argued that RA schedules elegantly describe a core life history trade‐off for plants. A focus on the allocation of energy by the plant at a given age or size allows RA schedules to be easily incorporated into a variety of process‐based plant growth and ecosystem models (e.g., Fisher et al. 2010; Falster et al. 2011; Scheiter et al. 2013). The division of energy between growth and reproduction is also the foundation of optimal energy models (Myers and Doyle 1983; Kozlowski 1992; Perrin and Sibly 1993; Reekie and Avila‐Sakar 2005; Miller et al. 2008).

Yet, our ability to systematically study the life history strategies of real plants and relate these to basic theory seems limited by the paucity of currently available data. We expect further integration of RA schedules into plant growth models will help clarify several empirical patterns. For example, growth rates among larger plants show only weak relationship to leaf traits (Wright et al. 2010) – this could be because substantial variation in RA among species veils the underlying effects of traits influencing mass production and deployment (Thomas 2010). Better empirical data on RA would also allow the wealth of predictions made by optimal energy models to be tested. For example, do physiological traits affecting growth and mortality rates have consequences for RA schedules, as theory would suggest (Pugliese and Kozlowski 1990) (Iwasa and Cohen 1989)? Miller et al. (2008) provides a rare exception, where empirical data was incorporated into an optimal energy model, convincingly showing that plant seed set, and hence RA, is strongly affected by insect attack. More data on RA schedules could also greatly improve our ability to model biogeochemical cycles and ecosystem food webs. The processes controlling allocation of carbon between different plant tissues has been identified as one of the most uncertain features of current biosphere models (De Kauwe et al. 2014). Whether carbon is allocated to building leaf, stem, or reproductive material has potentially large implications for predicted carbon fluxes and plant growth rates (Thomas 2011). For example, in a widely used model of regional carbon uptake and population dynamics, the ecosystem demography model (Moorcroft et al. 2001), a fixed fraction (0.3) of surplus energy is allocated to reproduction. Our results suggest this amount is lower than the maximum achieved by most species, but also that allocation varies substantially through ontogeny.

To address these key questions, make better comparisons and determine more generalities, data for RA schedules must be collected across many species using similar if not identical methods. Life history and functional traits must be measured for each species in order to determine how variation in these traits correlates with RA schedules. For decades, theoreticians have been using RA schedules as a fundamental evolvable trait (Myers and Doyle 1983; Iwasa and Cohen 1989; Kozlowski 1992). It's time we empiricists collected some data.

## Conflict of Interest

None declared.

## Appendix

For the studies where RA was not directly reported, we calculated the values as follows:

### Enright 1985

The manuscript provides data on net annual production across all tree sizes, dividing a plant into leaves, stem, underground tissues, and reproductive tissues. Total leaf number is constant among reproductively mature plants, and therefore, new leaf production is simply replacing shed tissues. RA is therefore calculated as yearly reproductive production divided by the sum of net annual production of stems, underground tissues, and reproduction. True RA values will be somewhat higher than those reported, especially for the oldest plants, as root turnover rates are not known.

### Genet 2010

The manuscript provides data on net annual increase in stem biomass, annual non‐structural carbohydrate production, and seed production. Reproductive biomass includes seed and cupule mass, but not other accessory costs. RA is calculated as seed production divided by the sum of increase in stem biomass, annual non‐structural carbohydrate production, and seed production. Data were collected in a significant mast year for *Fagus sylvatica*, such that RA averaged over several years would likely be lower. For *Fagus*, the accessory costs ignored should be small compared with seed and cupule mass, but their inclusion would increase RA.

### Hirayama 2004

The manuscript provides data on increase in “woody organ” biomass, total leaf biomass, and reproductive costs. Reproductive costs include accessory costs, including the number of flowers, aborted fruit, and mature fruit. The allometric equation they used to determine increase in “woody organ” biomass and leaf production are based on averages for an entire community and are not specific to the species they studied. They do not have data on plant age or yearly increase in leaf biomass, such that it is difficult to estimate proportion of total leaf biomass that replaces shed tissue and proportion that represents an increase in leaf biomass. As a rough approximate, we used average annual increase in dbh across all tree sizes (as there was no clear trend with tree size) to estimate difference in age between their 5 trees. We then divided the difference in leaf weight between trees by the difference in age to determine the annual increase in leaf mass. This rough calculation indicated that the majority of leaf biomass replaces the previous year's shed leaves, while a small fraction represents an increase in leaf biomass. RA is then calculated as reproductive production divided by a sum of increased wood biomass, increased leaf biomass, and reproductive production. This yields RA values up to 3 times greater than presented in their manuscript, although the pattern, a continued increase in RA with height is unchanged.

### Hirayama 2008

The manuscript provides data on increase in woody material, increase in leaf biomass, and reproductive investment. Reproductive investment includes mature fruit (including dispersal and protective material) as well as aborted flowers and fruits. RA is calculated as reproductive investment divided by the sum of increase in woody material, increase in leaf biomass, and reproductive investment. These species show a distinct pattern of biennial masting, so we averaged RA during a high and low mast year to determine the trees' actual allocation patterns. Note that the manuscript uses total leaf biomass, not incremental increase, in their calculation of RA, such that their estimates of RA are much lower than the ones we have used. The manuscript does not provide a threshold size or age for these species, only indicating they used a range of dbh values above 20 cm. *Quercus salicina* has a constant RA across this range, leading us to believe the smallest trees included are larger than trees at the “threshold size.” We therefore do not include a threshold size or RA for this species.

### Kohyama 1982

The data are from a figure in the manuscript. The manuscript does not indicate how “net reproductive effort” is calculated so we are uncertain whether it accounts for shed tissues. The reproductive investment component includes accessory tissues associated with the cone and seed formation. Masting occurs every 4 years, and numbers presented in the study are averaged across years to determine the long‐term RA.

### Pinero 1982

The manuscript includes data on net annual production of roots, trunk, live leaves, dead leaves (interpreted to mean shed leaves), seeds, and reproductive accessory tissues. RA is calculated as the sum of reproductive production divided by the sum of net annual production of roots, trunk, live leaves, and reproductive materials.

### Pritts 1985

The manuscript includes data on annual biomass production of vegetative and reproductive structures for a range of plant ages (their Fig. 3). Vegetative structures are divided into stems, roots, and leaves. Total new vegetative production is the sum of new stem production, new root production, and the increase in leaf biomass over the previous year. We use the increase in leaf production as *Vaccinium corymbosum* is a winter deciduous species and the other portion of leaf production offsets previously shed tissue. RA is calculated as reproductive production divided by the sum of reproductive and new vegetative production.

### Sakai 2003

The manuscript provides equations for increment increase of wood (R^2^H increment) and annual reproductive biomass for three populations of *Abies mariesii*. Neither annual leaf and root production, nor turnover of these structures, is known. RA could therefore not be calculated, although the possible shape of the RA curve could be. Using the equations provided and estimating a wood density of 0.5, annual wood production and reproductive production are determined. RA is then calculated as reproductive production divided by the sum of wood production and reproduction production. If a hypothetical increase in leaf area is included, shifting from double wood production in young plants to a small fraction of wood production in mature plants, the shapes of the curves of the middle and low population plants are unchanged, while the initial RA at the high site is much reduced and the RA across plant size is fairly unchanged.
